# Anti-phospholipase A2 Receptor Membranous Nephropathy Presenting as Common Geriatric Complaints: A Case Report

**DOI:** 10.7759/cureus.101983

**Published:** 2026-01-21

**Authors:** Wai Tak Victor Li, Kwan Hung Wu, Oi Yee Lin

**Affiliations:** 1 Department of Medicine, Queen Mary Hospital, Hong Kong, HKG; 2 Department of Medicine, Tung Wah Group of Hospitals Fung Yiu King Hospital, Hong Kong, HKG

**Keywords:** anti-phospholipase a2 receptor antibodies, chronic kidney disease, frailty, primary membranous nephropathy, rituximab

## Abstract

Anti-phospholipase A2 receptor (anti-PLA2R) antibodies are an established cause of primary membranous nephropathy (MN). However, typical symptoms of proteinuria, such as peripheral oedema and frothy urine, are not easily identified in the elderly, and other non-specific presentations, such as hypoglycaemia and fluid overload, may lead to delays in diagnosis.

We report a case of anti-PLA2R-associated MN in an elderly woman who presented with fluid overload and recurrent hypoglycaemia, both of which are common in this age group. Renal function and anti-PLA2R antibody titres improved substantially following treatment with rituximab. This case underscores the importance of early clinical suspicion and timely intervention in elderly patients in order to preserve residual renal function and optimise outcomes.

## Introduction

Membranous nephropathy (MN), a common cause of nephrotic syndrome, may occur secondary to malignancy or in association with autoantibodies. Anti-phospholipase A2 receptor (anti-PLA2R) antibodies are a well-established cause of primary MN [1]. In adults, nephrotic syndrome and MN typically present with overt proteinuria; however, these features may be less pronounced and more difficult to recognise in older individuals [2].

In this case report, we describe an elderly woman with underlying schizophrenia and metabolic syndrome who developed anti-PLA2R-associated MN, presenting with fluid overload and recurrent hypoglycaemia, both of which are frequent clinical findings in the geriatric population, which hindered early diagnosis.

## Case presentation

A 75-year-old Indonesian lady was institutionalised in a care facility since 2016 for treatment-resistant schizophrenia, requiring multiple oral and depot antipsychotics (including aripiprazole, benzhexol, olanzapine, and zuclopenthixol), which limited her premorbid status to being chairbound with only simple communication. She also has well-controlled hypertension and hyperlipidaemia, diagnosed after an episode of minor stroke in 2015.

She developed a gradual deterioration in renal function, starting in 2016. Serum creatinine rose from normal levels to around 150 µmol/L in late 2024, accompanied by an albumin drop from the normal range to 25 g/L during the same period. A spot urine protein-creatinine ratio (UPCR) was elevated at 4.33 mg/mgCr in 2022, but a definite diagnosis was not reached, as the patient and her family refused renal biopsy and other invasive procedures at that time.

Episodes of fluid overload led to repeated admissions in 2025; this was accompanied by a further decline in renal function, with serum creatinine rising to around 300 µmol/L and UPCR reaching 8.37 mg/mgCr in June 2025. She also developed recurrent hypoglycaemia, but serum insulin and C-peptide were normal. A 24-hour urine for protein was saved due to increasing proteinuria disproportionate to her baseline renal function and returned elevated at 2.52 g. Although in the sub-nephrotic range, workup for nephrotic syndrome revealed an increased anti-PLA2R antibody titre of 778.6 RU/mL via enzyme-linked immunosorbent assay (ELISA). Tumour markers, including CA 125 (56 U/mL) and CA 15.3 (34 U/mL), were elevated; however, family members opted not to undergo further malignancy workup or renal biopsy. Complement C3 and C4 levels were not decreased, at 135 mg/dL and 76 mg/dL, respectively. Hepatitis B surface antigen and core antibodies, as well as anti-hepatitis C and anti-human immunodeficiency virus antibodies, were not detected. No monoclonal bands were detected in the serum either.

After consultation with a renal physician, she was subsequently given two doses of rituximab at 1 g, which were well tolerated and associated with a satisfactory antibody response (down to 29.5 RU/mL four months post-treatment) and improvement in renal function (creatinine down to around 180 µmol/L and UPCR down to 5.13 mg/mgCr; see Table 1 for trends of laboratory values over time). Hypoglycaemic episodes were also less frequent after treatment.

**Table 1:** Trends of laboratory values over time Anti-PLA2R, anti-phospholipase A2 receptor; ELISA, enzyme-linked immunosorbent assay; UPCR, urine protein-creatinine ratio

	Normal range	Baseline (2010)	First deranged renal function (November 2024)	Before diagnosis (June 2025)	After rituximab (October 2025)
Serum creatinine (µmol/L)	49 - 82	75	126	312	181
Anti-PLA2R antibody titre by ELISA (RU/mL)	<20	Not tested	Not tested	778.6	29.5
UPCR (mg/mgCr)	<0.09	Not tested	2.92	8.37	5.13

## Discussion

Anti-PLA2R antibodies are present in approximately 70% of patients with primary MN. These antibodies form subepithelial immune complexes along the glomerular basement membrane, driving podocyte injury and nephrotic-range proteinuria. These transmembrane PLA2R are highly expressed among podocytes, but their exact role in glomerular filtration is not clearly delineated [1].

Clinically, patients with anti-PLA2R MN present with features of nephrotic syndrome, including peripheral oedema from hypoalbuminemia, hyperlipidaemia, and heavy proteinuria - some of which were also identified in our patient. Hypoglycaemia can occur in nephrotic syndrome through several mechanisms, including urinary loss of insulin-binding proteins, which leads to increased levels of bioactive insulin. Additionally, progression to chronic kidney disease (CKD) can cause impaired renal insulin clearance and reduced hepatic gluconeogenesis [3]. The disease activity of anti-PLA2R MN can be monitored by serum antibody titre, as a higher baseline antibody titre correlates with disease severity, including lower serum albumin, higher proteinuria, lower glomerular filtration rate, and higher serum creatinine levels [4]. Current evidence suggests that renal biopsy is not essential for making a diagnosis [5]; however, tissue staining may be present before the emergence of circulating antibodies, thereby allowing earlier diagnosis, and could persist after bloodstream antibodies subside [6]. Compared with traditional immunosuppressive therapies, such as cyclosporine, rituximab is an effective and safe drug for anti-PLA2R MN, inducing serological responses and reducing proteinuria, which correlate with long-term renal outcomes [7]. In fact, without proper treatment, 35%-40% of patients with anti-PLA2R MN will progress to end-stage renal failure [2].

Fluid overload and hypoglycaemia are relatively common complaints among the frail elderly. Reduced cardiorenal reserve, diminished homeostatic responses, and a high prevalence of CKD and heart failure increase the risk of volume overload. Concurrently, cognitive impairment, psychiatric comorbidities, and functional decline contribute to malnutrition and poor intake, leading to hypoglycaemia. Patients with psychiatric illnesses have a two-fold higher risk of malnutrition compared to the general population [8]. The ubiquity of these geriatric syndromes can sometimes obscure early identification of specific renal pathologies [9], as evidenced by the higher median serum creatinine level at diagnosis among the elderly [10]. In fact, older patients who have nephrotic syndrome often present with more vague symptoms, which may delay diagnosis [11]. Common symptoms of nephrotic syndrome, such as frothy urine from proteinuria or peripheral oedema, are also commonly misdiagnosed as heart failure in these patients due to their age [12]. Malignancies, such as those of the lung and gastrointestinal tract, should be screened for in elderly patients with MN, as they are common secondary causes [13].

## Conclusions

This case highlights that geriatricians must watch for serious renal disease in older adults with non-specific symptoms. In our case, earlier recognition and a thorough workup for MN would have enabled prompt disease-modifying therapy. Regular metabolic panel and urinalysis checks also lead to earlier diagnosis. Early referral to nephrologists can prompt timely treatment with immunosuppressive agents, which improves long-term renal outcomes in frail patients with anti-PLA2R MN.
